# A New Hybrid Reinforcement Learning with Artificial Potential Field Method for UAV Target Search

**DOI:** 10.3390/s25092796

**Published:** 2025-04-29

**Authors:** Fang Jin, Zhihao Ye, Mengxue Li, Han Xiao, Weiliang Zeng, Long Wen

**Affiliations:** 1 College of Electrical Engineering, Naval University of Engineering, Wuhan 430033, China; 2 National Key Laboratory of Electromagnetic Effect and Security on Marine Equipment, China Ship Development and Design Center (CSDDC), Wuhan 430000, China; 3 School of Mechanical Engineering and Electronic Information, China University of Geosciences, No. 388 Lumo Road, Wuhan 430074, China

**Keywords:** reinforcement learning, target search, artificial potential field, unmanned aerial vehicle

## Abstract

Autonomous navigation and target search for unmanned aerial vehicles (UAVs) have extensive application potential in search and rescue, surveillance, and environmental monitoring. Reinforcement learning (RL) has demonstrated excellent performance in real-time UAV navigation through dynamic optimization of decision-making strategies, but its application in large-scale environments for target search and obstacle avoidance is still limited by slow convergence and low computational efficiency. To address this issue, a hybrid framework combining RL and artificial potential field (APF) is proposed to improve the target search algorithm. Firstly, a task scenario and training environment for UAV target search are constructed. Secondly, RL is integrated with APF to form a framework that combines global and local strategies. Thirdly, the hybrid framework is compared with standalone RL algorithms through training and analysis of their performance differences. The experimental results demonstrate that the proposed method significantly outperforms standalone RL algorithms in terms of target search efficiency and obstacle avoidance performance. Specifically, the SAC-APF hybrid framework achieves a 161% improvement in success rate compared to the baseline SAC model, increasing from 0.282 to 0.736 in obstacle scenarios.

## 1. Introduction

The autonomous navigation of unmanned aerial vehicles (UAVs) has attracted widespread attention due to its potential in various applications such as search and rescue [1,2] surveillance [3], and environmental monitoring [4]. To achieve efficient autonomous navigation and target searching in complex and dynamic environments [5,6], UAVs, colloquially known as drones, need to make real-time autonomous decisions. This requires UAVs to perform path planning, avoid obstacles, and adjust flight strategies flexibly in real time. As UAV technology advances, achieving full-process autonomy from environmental perception to autonomous decision-making has become crucial for UAVs to accomplish efficient target search and real-time obstacle avoidance in complex environments. This represents a core challenge that urgently needs to be addressed in the field of UAV autonomous navigation.

Traditional target searching methods often rely on heuristic algorithms [7,8] or model-based [9] path planning. Fu et al. [8] enhanced the A* algorithm by incorporating adaptive neighborhood search and dynamic weighting, achieving a 25.4% improvement in mobile robot navigation efficiency. Huang et al. [9] proposed an improved RRT algorithm using dynamic density gradient sampling and path reconstruction to reduce redundant sampling in inefficient regions, improving UAV path planning efficiency and path quality by approximately 25% compared to traditional RRT. However, these methods typically suffer from high computational complexity and poor real-time performance when dealing with obstacle avoidance in dynamic environments. Recently, reinforcement learning (RL) has shown significant potential in UAV navigation and path planning [10], which has attracted extensive research attention. Its core advantage lies in dynamically optimizing decision-making strategies through continuous interaction with the environment, which can improve target search efficiency greatly, especially in complex and dynamic environments.

However, RL’s application in large-scale environments for target searching and obstacle avoidance faces significant challenges. These include slow convergence, low computational efficiency, and limited effectiveness in high-dimensional state spaces, particularly in situations in which there exist many local obstacle avoidances. To improve performance, many studies have attempted to explore the integration of RL with other algorithms to enhance its obstacle avoidance and path planning capabilities. Varma and Kumari [11] proposed a fuzzy rule-based deep learning model combined with swarm intelligence for efficient obstacle avoidance in UAVs, achieving a high accuracy of 98.62% in handling both static and dynamic obstacles. Bo et al. [12] developed an improved Q-learning algorithm based on flower pollination and tabularization methods to address the issue of convergence speed and dynamic response time in complex environments. Meanwhile, the Artificial Potential Field (APF) [13] method constructs virtual force fields, offering simple yet efficient obstacle avoidance, performing particularly well in static environments. By integrating this method with other algorithms, UAV navigation efficiency in complex environments can be significantly improved, optimizing obstacle avoidance behaviors and allowing more precise and real-time decision-making. However, how to effectively combine RL-based global optimization with APF-driven local obstacle avoidance still requires further investigation.

In this study, a hybrid framework combining RL and APF is proposed to enhance UAV navigation performance in dynamic environments. The RL component serves as the global strategy planner, responsible for target search and optimal path planning over a large area, thus enabling adaptability to environmental uncertainties. Meanwhile, the APF acts as the local strategy executor, offering real-time guidance for obstacle avoidance and ensuring safe and efficient navigation through complex environments. This fusion approach leverages RL’s global optimization strengths while utilizing the APF’s geometric navigation capabilities for local obstacle avoidance, effectively improving target search efficiency and ensuring safe, real-time path planning performance. To evaluate the performance of this hybrid approach, a set of controlled simulation scenarios was designed with static obstacles, offering a clear testing environment for analyzing the RL–APF framework’s obstacle avoidance and target search capabilities. Through comparative simulation experiments with base RL algorithms, the performance of the hybrid RL–APF framework was evaluated in static obstacle environments, focusing on target search efficiency and obstacle avoidance capability. The results demonstrate that the hybrid framework achieves significant improvements in target search success rates compared to using RL algorithms alone. Specifically, SAC–APF achieved a 161% improvement over the base SAC model’s 0.282, reaching a level of 0.736. This highlights the hybrid framework’s significant advantages in both target search and obstacle avoidance.

The paper is organized as follows. The related works are presented in Section 2. The problem definition is presented in Section 3. The proposed method is presented in Section 4. The experiment results and discussion are presented in Section 5. Finally, the conclusion is summarized in Section 6.

## 2. Related Works

UAVs have been widely applied in fields such as surveillance, rescue, environmental monitoring, and transportation [14,15]. Navigation and path planning are essential for UAV autonomy, and RL effectively optimizes UAV decision-making through environmental interaction, enhancing task efficiency. Numerous studies have explored the application of RL in UAV navigation. Zhang et al. [16] proposed a Deep Reinforcement Learning (DRL)-based autonomous UAV navigation method using the Twin Delayed Deep Deterministic Policy Gradients (TD3) algorithm. By incorporating changes in environmental observations, the method enhances UAV navigation capabilities in multi-obstacle, random, and dynamic environments. Sheng et al. [17] optimized UAV flight efficiency and navigation performance in high-density, high-dynamic environments through state space representation and dynamic reward function. Hussein Samma et al. [18] introduced an improved DRL method combining RL and self-supervised learning for autonomous visual navigation of UAVs in dynamic environments, significantly enhancing navigation performance and learning efficiency. Liu et al. [19] proposed a hierarchical RL algorithm based on an attention mechanism, improving UAV autonomous navigation by incorporating average estimation functions, recurrent networks, temporal attention mechanisms, and a hierarchical framework. Zhang et al. [20] proposed an RL-based Cooperative Navigation algorithm (RL–CN), combining enhanced reinforcement learning methods, reward shaping, staged policy adjustment, and prioritized experience replay strategies to effectively improve cooperative navigation capabilities of multiple UAVs in dense obstacle and dynamic environments.

The APF method is an intuitive and efficient path planning approach that provides real-time navigation capabilities for UAVs, making it widely used in autonomous flight tasks. Many studies have explored its application in UAV path planning. Cezary Kownacki [21] proposed a quadrotor UAV moving target trajectory tracking method based on APF, which synthesizes control velocity vectors to enable the UAV to track dynamic targets or complex trajectories in real time while maintaining low tracking errors. Hao et al. [13] addressed the problems of local minima, unreachable targets, and unreasonable obstacle avoidance in traditional APF by incorporating collision risk assessment and virtual sub-goals. Zhang et al. [22] solved the local optimum problem in traditional APF by adding collinear force deflection angles, enhancing the obstacle avoidance capability; they also developed an APF-based collision avoidance strategy to improve the flight safety and efficiency of UAV formations in dynamic and complex environments. Han et al. [23] integrated an improved Grey Wolf Optimizer (GWO) and APF algorithm, introducing nonlinear adjustment strategies and optimized individual position update strategies to balance global and local search capabilities, reducing the possibility of becoming trapped in local optima. This approach is suitable for UAV path planning tasks in disaster rescue scenarios.

UAV navigation and path planning are core issues for their autonomous flight, with RL being suitable for navigation in dynamic and unknown environments, while APF is more suitable for obstacle avoidance in static environments. Many researchers have proposed improved solutions, and hybrid approaches that combine global path planning and other optimization techniques are becoming a growing trend in research. Kong et al. [24] introduced an optimized path planning algorithm based on Deep Q-Networks (DQN) and APF, called B-APFDQN, which enhances training efficiency through multi-output neural networks and SA-ε-greedy algorithms, avoids local optima, and results in shorter, smoother paths. Li and Wu [25] proposed an improved Deep Deterministic Policy Gradient algorithm for UAV ground target tracking. By combining the line-of-sight and APF reward functions, action penalty terms, multi-UAV cooperative tasks, and Long Short-Term Memory networks for processing historical observations, they improved target tracking and obstacle avoidance performance. Li et al. [26] proposed an improved Dueling DQN algorithm, which combines prioritized experience replay and APF intervention strategies. This approach effectively addresses the issues of low learning efficiency, slow convergence speed, and slow inference speed in traditional DQN algorithms for robot path planning, significantly improving the network’s convergence performance and path planning quality. Xi et al. [27] presented a framework that combines APF and DRL for obstacle avoidance. By adjusting target positions and incorporating velocity information, their method effectively reduces path oscillations and enhances obstacle avoidance performance for quadrotor UAVs.

As the related literature shows, both RL and the APF method provide effective solutions for UAV navigation. In this work, an RL–APF hybrid algorithm framework, which combines the real-time responsiveness of APF-based obstacle avoidance control with the adaptive decision-making ability of RL, is proposed to address the issues of low obstacle avoidance efficiency and unstable navigation in target search tasks in complex environments.

## 3. Problem Definition

This section primarily introduces the task description of UAV target search, the environment setup, and the definition of the UAV model, providing the foundational concepts for the subsequent algorithm framework design and experimental simulations.

### 3.1. Task Description

**Figure 1:**
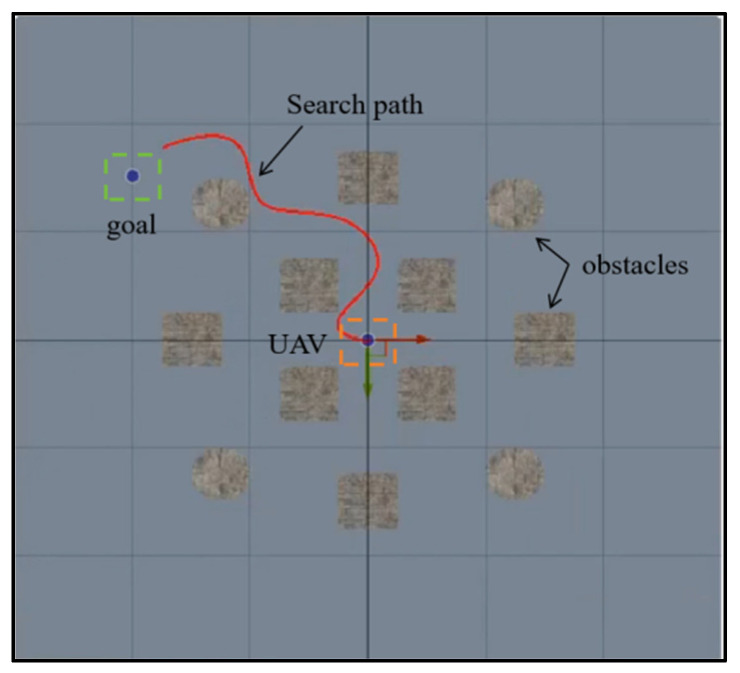
The process of the UAV target search.

The UAV starts from the center of the flight area and perceives the surrounding environment’s state information through depth images captured by camera sensors in order to avoid obstacles and reach the target location. To enhance the randomness of the search scenario, the target’s location is randomly generated. Meanwhile, the success or failure of the target search mission is determined by whether the UAV can successfully reach the target and avoid obstacles. If the UAV’s distance to the target is smaller than a predefined threshold, the task is considered successful. The task fails if the UAV collides with an obstacle or leaves the flight area. The process of the UAV target search mission is shown in Figure 1.

The performance of the target search task is evaluated through four key metrics. (1) Average Reward. This is calculated as the mean cumulative reward across the entire testing period to reflect the overall decision-making quality. (2) Success Rate. This is defined as the percentage of episodes in which the drone successfully locates the target to represent mission accomplishment efficiency. (3) Loss Rate. This is computed as the percentage of episodes where the drone fails to reach the target due to either obstacle collisions or boundary violations within the predefined episode limit to characterize system robustness. (4) Task Completion Steps. This is recorded as the average number of steps required for successful missions to evaluate search efficiency.

### 3.2. Environment

**Figure 2:**
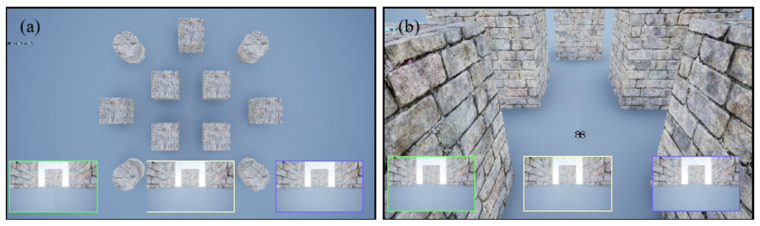
Simulation environment. (**a**) Top-down view of the entire simulation environment in UE4, showing the layout and surroundings. (**b**) Front view from the drone’s perspective during flight, with the rectangular box representing the RGB image captured by the onboard camera.

The simulation environment is shown in Figure 2, where the UAV’s flight area is set as a 120 × 120 three-dimensional space, used to simulate the UAV’s flight mission. The environment state consists of the UAV’s current position, the target position, and the positional information of multiple obstacles. The UAV’s position is denoted as $P_{u}=(x,y,z)$, and the target position as $P_{g}=(x_{g},y_{g},z_{g})$. To simplify the target search task, both the UAV and the target are constrained to a certain altitude. The environment contains positional information of multiple obstacles, and the set of obstacles is denoted as $O=\{P_{o}^{1},P_{o}^{2},\ldots ,P_{o}^{n}\}$, where $P_{o}^{i}$ represents the three-dimensional coordinates of the i-th obstacle. For cylindrical obstacles, their position is given by $P_{o}^{i}=(x_{i},y_{i},z_{i})$, and the boundary is defined by their radius $r_{i}$. For cuboid-shaped obstacles, the boundary is defined by the edge length $l_{i}$. The height of all obstacles is h.

### 3.3. UAV Model

**Figure 3:**
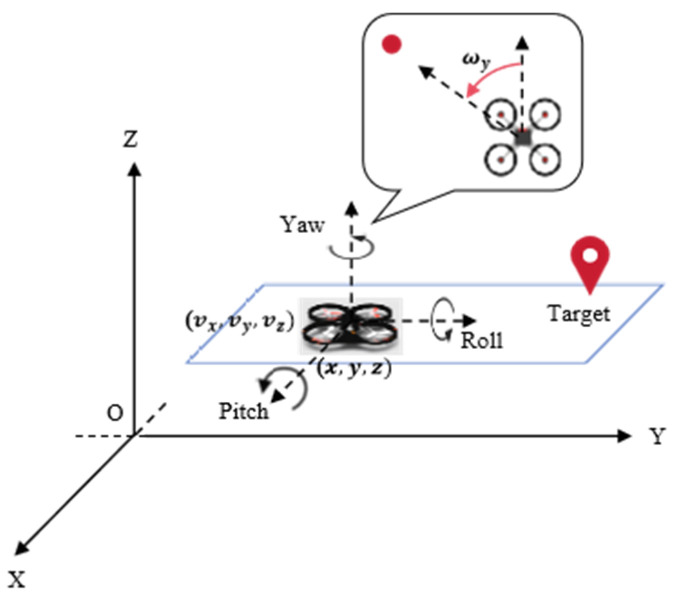
The UAV flight parameters.

This study is based on the quadrotor UAV model, as shown in Figure 3. The position information of the UAV is denoted as $P_{u}=(x,y,z)$, and the linear velocity of the UAV is denoted as $v_{u}=(v_{x},v_{y},v_{z})$. Due to the height limitation in the planar space, and for simplicity in modeling, only the yaw angular velocity of the UAV is considered, denoted as $\omega_{y}$. The UAV’s motion is influenced by the thrust $F_{T}$ and the moment M, with the thrust direction related to the speed of the four motors. The kinematic equation is shown in Equations (1) and (2), where m is the mass of the quadrotor, g is the acceleration due to gravity, and $F_{T}$ is the thrust produced by the quadrotor.

(1) $$\dot{P}=v$$

(2) $$\dot{v}=g+F_{T}/m$$

The UAV platform is equipped with a forward and dual-side tri-vision system [28], offering a 210° horizontal field of view. This system enables real-time environmental perception, supporting autonomous navigation and dynamic obstacle avoidance capabilities.

## 4. Methodology

**Figure 4:**
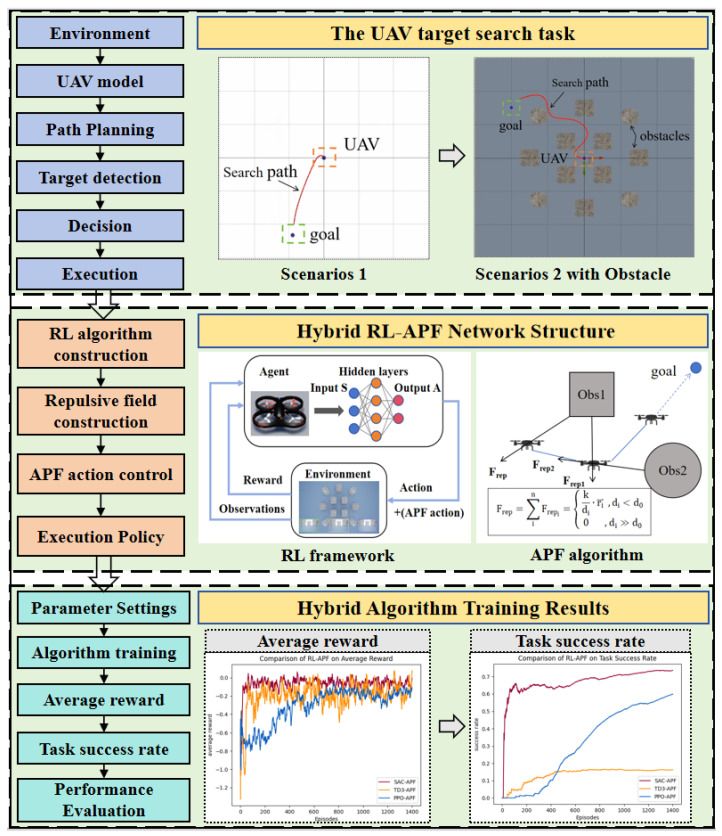
Hybrid RL–APF for UAV target search.

To achieve efficient target search for drones in complex and dynamic environments, a hybrid framework is proposed in this research. As illustrated in Figure 4, the key components of this method, including the hybrid RL–APF network structure and the results of the hybrid algorithm training, are presented and discussed in detail in the following sections. Based on the problem definition of the UAV target search task, this section focuses on the proposed framework.

### 4.1. Reinforcement Learning Framework

RL is a machine learning method aimed at learning the optimal strategy through interaction with the environment. In reinforcement learning, an agent makes a series of decisions in the environment, with each decision receiving a reward signal that reflects the contribution of the agent’s behavior to the goal. The agent updates its strategy by observing the reward signals to maximize the long-term cumulative reward. The basic elements of the reinforcement learning process include the agent, environment, state, action, reward, and policy, with a focus on the design of the state space, action space, and reward function. In this study, the definition of RL is as follows.

#### 4.1.1. State Space

To help the UAV better understand and perceive the environment, a set of state variables is defined in the state space to comprehensively describe the relationship between the UAV and the target, the UAV’s own motion state, and its position in the two-dimensional space. The state space is shown in Equation (3), where S represents the state, $P_{u}=(x,y,z)$ represents the current position coordinates of the UAV, $d_{goal}=\sqrt{{\left(x-x_{g}\right)}^{2}+{\left(y-y_{g}\right)}^{2}}$ represents the 2D distance between the UAV and the target, ∆θ is the relative yaw angle within the range (−π,π), and $d_{i}=\sqrt{{\left(x-x_{{obs}_{i}}\right)}^{2}+{\left(y-y_{{obs}_{i}}\right)}^{2}}$ represents the distance between the UAV and the obstacle.

(3) $$S=[P_{u},d_{goal},∆\theta ,v_{u},\omega_{y},d_{i}]$$

#### 4.1.2. Action Space

The design of the action space employs a velocity control method, primarily consisting of horizontal velocity and yaw angular velocity. The specific action space is shown in Equation (4), where A represents the action, $v_{xy}=\sqrt{v_{x}^{2}+v_{y}^{2}}$ represents the UAV’s speed in the horizontal plane, and $\omega_{y}$ is used to control the steering of the UAV.

(4) $$A=[v_{xy},\omega_{y}]$$

#### 4.1.3. Reward Function

The design of the reward function is based on the environmental state and the agent’s behavior. It considers multiple factors, including target proximity, position penalty, action control, and obstacle avoidance penalties, to guide the UAV in making efficient decisions and optimizing its behavior during the search task.

The target proximity reward $r_{distance}$ is based on the distance between the UAV and the target. When the UAV moves toward the target, the reward increases; conversely, it decreases when moving away from the target. The reward calculation formula is shown in Equation (5). $d_{-1}$ represents the distance at the previous time step, and $d_{0}$ represents the distance at the current time step.

(5) $$r_{distance}=\frac{d_{-1}-d_{0}}{d_{goal}}$$

The position penalty $r_{position}$ is based on the UAV’s current position deviation relative to the target position. It considers the UAV’s deviation in both the XY plane and along the *Z*-axis. The specific calculation formula is shown in Equation (6), where $d_{x}$ is the horizontal distance between the UAV and the target, D is the maximum allowable horizontal distance, $z\;and\;z_{g}$ are the current and target heights, H is the maximum allowable height difference, and $\lambda_{x}$ and $\lambda_{z}$ are the weight coefficients for the horizontal and vertical deviations, respectively.

(6) $$r_{position}=\lambda_{x}\frac{d_{x}}{D}+\lambda_{z}\frac{\left|z-\right.\left.z_{g}\right|}{H}$$

The action control penalty $r_{action}$ considers the UAV’s heading rate, vertical velocity, and height error and is calculated as shown in Equation (7), where $\omega_{y}$ is the heading rate, $v_{z}$ is the vertical speed, $\omega_{max}$ is the maximum heading rate, $v_{max}$ is the maximum vertical speed, and $\beta_{y}$, $\beta_{v}$, and $\beta_{z}$ are the respective weight coefficients for the heading rate, vertical velocity, and height error.

(7) $$r_{action}=\beta_{y}\frac{\left|\omega_{y}\right|}{\omega_{max}}+\beta_{v}\frac{\left|v_{z}\right|}{v_{max}}+\beta_{z}\frac{\left|z-\right.\left.z_{g}\right|}{H}$$

To prevent the UAV from colliding with obstacles, an obstacle avoidance penalty $r_{obstacle}$ is added. When the UAV’s distance to the nearest obstacle falls below a preset threshold, the penalty value increases, guiding the UAV to maintain a safe distance. The specific expression is shown in Equation (8), where $d_{min}$ represents the UAV’s minimum distance to the nearest obstacle, and $d_{safe}$ is the safety distance threshold, representing the safe distance between the UAV and an obstacle.

(8) $$r_{obstacle}=max\left(0,1-\frac{d_{min}}{d_{safe}}\right)$$

Considering the above factors, the final reward function is calculated as shown in Equation (9), where $\alpha_{p}$, $\alpha_{a}$, and $\alpha_{o}$ are the corresponding weights for position penalty, action penalty, and obstacle avoidance penalty, respectively.

(9) $${R=r}_{distance}-\alpha_{p}r_{position}-\alpha_{o}r_{obstacle}-\alpha_{a}r_{action}$$

After the task is completed, if the UAV successfully reaches the target location, a reward is given: $r_{r}=10$. If the UAV leaves the operational space, a penalty is applied: $r_{o}=-10$. If the UAV collides with an obstacle, a penalty is applied: $r_{o}^{\prime }=-20$. By integrating these components into the reward function, the UAV is guided to complete the target search task, achieving efficient autonomous navigation and decision-making capabilities.

#### 4.1.4. Soft Actor-Critic

Soft Actor-Critic (SAC) [29] is a policy-based RL designed to directly select actions by optimizing the policy, suitable for continuous action spaces. Unlike Proximal Policy Optimization (PPO) [30] and Twin Delayed Deep Deterministic Policy Gradient (TD3) [31], PPO uses a trust region optimization-based policy gradient method to ensure stability during the update process, while TD3 addresses the instability of Q-value estimation by introducing techniques such as double Q-networks, target smoothing, and delayed updates. One of the key innovations of SAC is the introduction of the maximum entropy principle, which promotes exploration and improves learning efficiency. It not only optimizes the expected reward but also encourages randomness in the policy’s action selection, enhancing exploration. At the same time, SAC combines value-based methods and policy-based methods, adopting a “soft” policy update approach, making it excel in goal-directed tasks.

SAC uses two Q-networks (Q1 and Q2), and by taking the minimum value, it reduces overestimation bias, thus improving the stability and performance of training, as shown in Algorithm 1. Additionally, it uses a deterministic target policy and a stochastic policy update method, effectively balancing exploration and exploitation. When optimizing the policy, SAC not only focuses on expected rewards but also adds an entropy term to make the policy more random. The objective function is shown in Equation (10), where α is the hyperparameter that controls the entropy weight, adjusting the balance between exploration and exploitation.

(10) $$J\left(\pi \right)={Ε}_{\left(s,a\right)\sim \rho_{\pi }}\left[Q\left(s,a\right)-\alpha \log\left(\pi \left(a\left|s\right.\right)\right)\right]$$

**Algorithm 1**: SAC training algorithmInitialize the policy parameters θ and value networks $Q\left(s,a\right)$ Initialize the target Q network $Q_{\mathrm{target}}$ Initialize the entropy coefficient α **For** each episode e = 1 → E:  Initialize the state s from the environment  **For** each time step t = 1 → T:    Use the Actor’s policy network to select an action a    Execute action a, observe the reward r, and the next state s′    Store the transition in the experience replay buffer $\left(s,a,r,s^{\prime }\right)$    Sample a batch of data from the experience replay buffer    Compute the target value $Q_{\mathrm{target}}=r+\gamma V\left(s^{\prime }\right)$ and TD error $\delta =Q_{\mathrm{target}}-V\left(s\right)$    Minimize the TD error to update the value network $Q_{critic}\left(s,a\right)$    Adjust the policy by maximizing the target value $Q_{\mathrm{target}}$    Calculate the policy loss $L_{actor}=\alpha \cdot log(\pi (a|s))+Q_{actor}(s,a)$    Update the policy network $Q_{actor}(s,a)$    Update the entropy coefficient α to balance exploration and exploitation    Soft-update the target Q network and target value network parameters     Update the current state s to the next state $s^{\prime }$  **end for** **end for**

**Figure 5:**
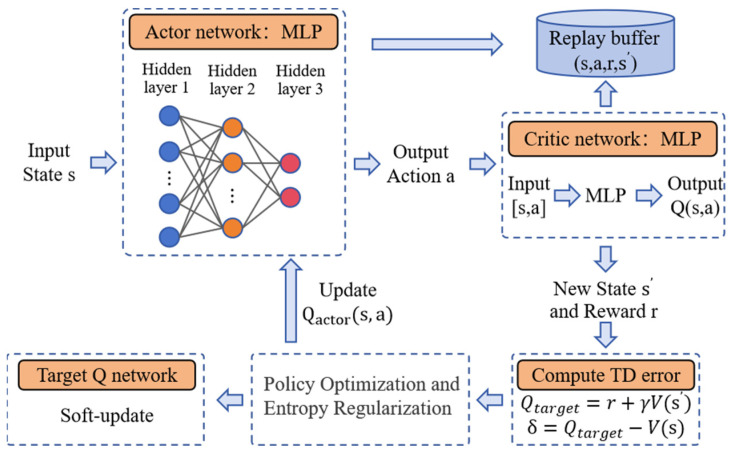
SAC algorithm training framework diagram.

The training process of the SAC algorithm is shown in Figure 5. In terms of the network architecture, a Multilayer Perceptron (MLP) [32] is used as the core model for the reinforcement learning policy. This network structure includes three hidden layers with 64, 32, and 16 neurons, respectively, and uses the hyperbolic tangent activation function. This architecture is capable of handling the nonlinear features in the task and assists the UAV in performing efficient target search and navigation in complex environments.

### 4.2. Hybrid RL–APF Method

To improve the performance of RL and APF in complex environments, this research proposes a hybrid method that combines RL and APF to form a framework capable of efficiently addressing target searching tasks in dynamic environments. The core idea of this hybrid approach is to incorporate the guiding role of artificial potential fields within the reinforcement learning framework, thereby optimizing the navigation and obstacle avoidance capabilities of the UAV.

#### 4.2.1. Artificial Potential Field

The APF method constructs an artificial virtual potential field to plan the UAV’s motion path, guiding the UAV to avoid obstacles and reach the target point during its movement. It is widely applied in the fields of UAV motion control and intelligent navigation. In UAV target searching tasks, the target location is unknown, so only a repulsive force field between the UAV and obstacles needs to be constructed. In this repulsive force field, the UAV experiences a repulsive force from obstacles, with the magnitude of this force inversely proportional to the distance between the UAV and the obstacle. The specific expression is shown in Equation (11), where k is the repulsive force intensity coefficient, $\overset{⃑}{r_{i}}$ is the direction vector pointing away from the obstacle, and $d_{0}$ is the distance threshold at which the repulsive force becomes effective.

(11) $$F_{rep}=\sum_{i}^{n}F_{{rep}_{i}}=\left\{\begin{array}{cc} \frac{k}{d_{i}}\cdot \overset{⃑}{r_{i}}, & d_{i}<d_{0}\\ 0\;\;\;\;\;\;\;\;, & d_{i}\gg d_{0} \end{array}\right.$$

**Figure 6:**
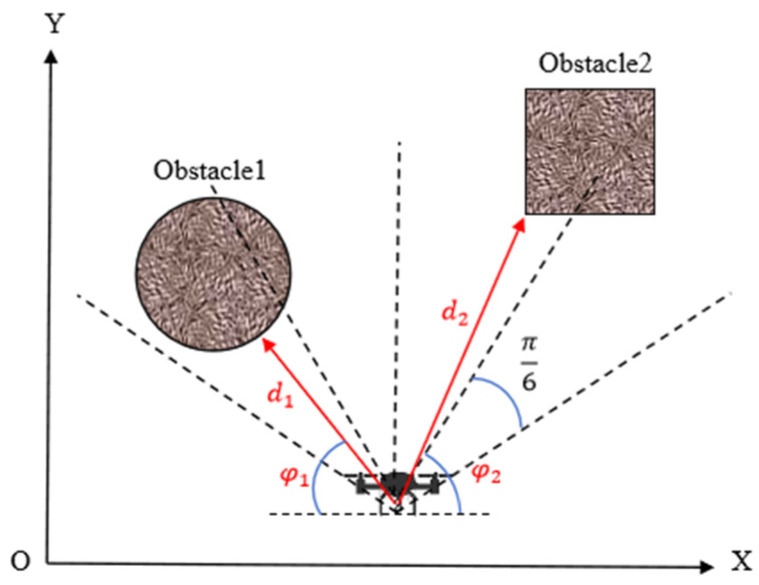
The relative spatial relationship between the UAV and the obstacles.

Based on the UAV’s visual perception system, the forward detection area is equally divided into $\frac{\pi }{6}$ sector partitions. The minimum Euclidean distance between the obstacle point cloud in the depth image of each partition and the UAV is calculated, thereby obtaining the closest distance $d_{i}$ and direction angles $\varphi_{i}$ between the UAV and multiple obstacles, as specifically illustrated in Figure 6. The repulsive force components in the x and y directions are as shown in Equations (12) and (13).

(12) $$X_{rep}=\sum_{i}^{n}X_{{rep}_{i}}=\frac{k}{d_{i}}\cdot \cos\varphi_{i}$$

(13) $$Y_{rep}=\sum_{i}^{n}Y_{{rep}_{i}}=\frac{k}{d_{i}}\cdot \sin\varphi_{i}$$

By synthesizing the repulsive forces generated by all obstacles, the UAV can dynamically adjust its motion path to avoid obstacles while maintaining its search task toward the target point.

#### 4.2.2. Hybrid Algorithm Structure

**Figure 7:**
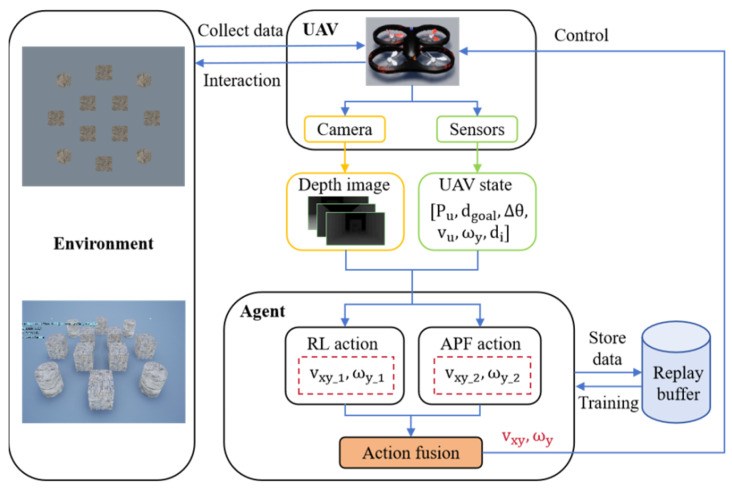
RL–APF hybrid decision-making framework.

Using the hybrid RL and APF method, efficient target search and obstacle avoidance for UAVs can be achieved in complex environments. Reinforcement learning is responsible for autonomously learning the optimal decision-making strategy in dynamic environments, guiding the UAV to choose appropriate actions through a reward mechanism to complete the target search task. The artificial potential field method provides real-time obstacle avoidance capability by simulating a virtual force field to guide the UAV in avoiding obstacles. The core idea of the hybrid algorithm structure is as follows. First, the APF is used to compute the obstacle avoidance force field based on environmental obstacles. Then, the global path control signal generated by the RL strategy is combined. Finally, the control commands of both are weighted and fused to generate the final flight command. The specific process is shown in Figure 7.

The control signals of APF and RL are combined using a weighted fusion approach, where different weighting strategies are applied to $v_{xy}$ and $\omega_{y}$. Within a certain range, the weights are determined through cross-experimental validation to effectively balance local obstacle avoidance and global path planning. The weighting formula for the $v_{xy}$ can be expressed as shown in Equation (14), where $\varepsilon_{1}=0.7$ indicates that $v_{xy}$, controlled by the RL strategy, dominates.

(14) $$v_{xy}=\varepsilon_{1}v_{xy\_RL}+\left(1-\varepsilon_{1}\right)v_{xy\_APF}$$

The weighting formula for $\omega_{y}$ can be expressed as shown in Equation (15), where $\varepsilon_{2}=0.3$ indicates that in terms of heading adjustment, the APF obstacle avoidance control action has a larger weight.

(15) $$\omega_{y}=\varepsilon_{2}\omega_{y\_RL}+\left(1-\varepsilon_{2}\right)\omega_{y\_APF}$$

By combining both approaches, reinforcement learning optimizes the UAV’s global search strategy, while the artificial potential field handles local obstacle avoidance issues, thus improving the UAV’s navigation efficiency and safety in complex environments.

## 5. Experimental Setup

### 5.1. Simulation Platform

This study constructs a high-fidelity simulation platform for testing autonomous UAVs in complex environments. The simulation environment is based on Unreal Engine 4 (UE4), with AirSim 1.6.0 used as a plugin for UE4 to build the UAV’s dynamics model and physics engine. The simulation platform is integrated with OpenAI Gym 1.7.3, providing a good interactive interface for training RL algorithms.

To ensure the stability, safety, and control accuracy of the UAV’s flight, the following constraints are applied to its flight parameters: a time step of 0.1, a maximum horizontal acceleration of 2.0, a horizontal speed range of 0.5 to 3.5, and a maximum yaw rate limit of 30.0. RL algorithm training and hybrid algorithm training are conducted in two different scenarios: one without obstacles and one with obstacles. The performance of different algorithms is evaluated through average reward and task success rate. To ensure the stability and convergence of algorithm training, the key hyperparameters are configured as follows: discount factor γ=0.99, learning rate α=0.0001, learning starts = 2000, experience replay buffer size = 50,000 samples, batch size = 512, network update frequency = every 100 time steps, and gradient steps = 100 per update.

### 5.2. Scenario 1: Target Search

**Figure 8:**
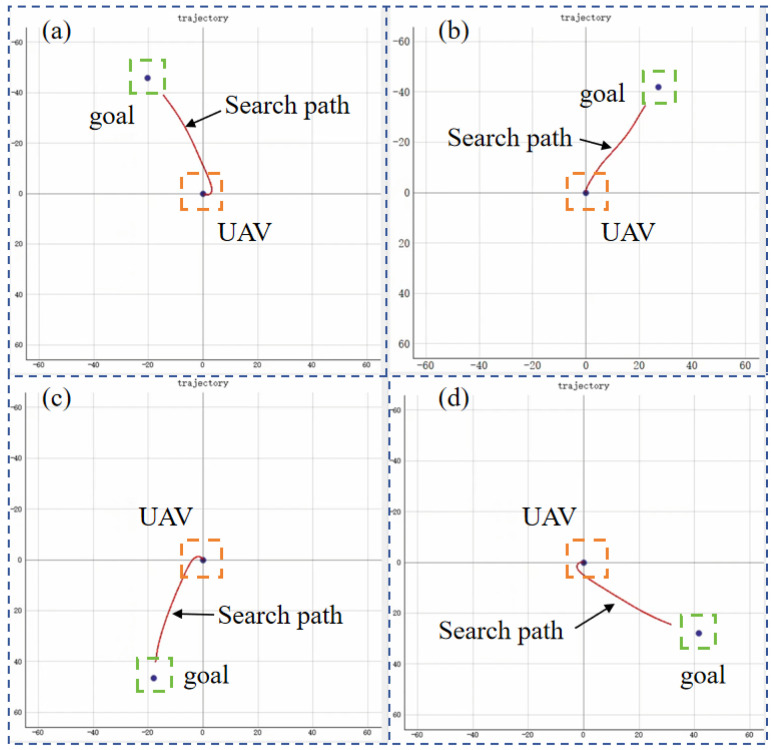
The search process for different target positions. (**a**–**d**) are selected from four different episodes out of a total of 1400 episodes, targeting search tasks with randomly generated target positions in an obstacle-free scenario. The red lines represent the UAV’s search trajectory, starting from the center point. The orange square represents the UAV, and the green square represents the target.

This scenario is the target search without any obstacles. Three RL algorithms are adopted to train the UAV for target searching, and they are PPO, SAC, and TD3. Targets are randomly generated to evaluate the exploration efficiency and stability of the reinforcement learning algorithms. The search process for different target positions is shown in Figure 8.

**Figure 9:**
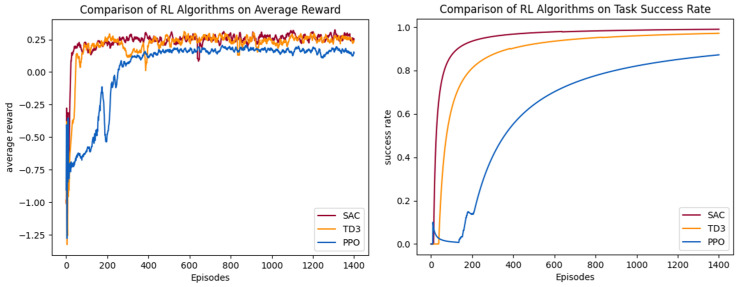
The training results of different RL algorithms in Scenario 1.

The average reward curves and task success rate curves for the three different algorithms are shown in Figure 9. In the early stages of training, the UAV has not yet learned an effective policy, leading to unstable performance in the environment, large fluctuations in reward, and a very low task success rate. As the number of training episodes increases, the average reward gradually converges, and the success rate improves to its peak.

The comparative analysis of the experimental results demonstrates that both SAC and TD3 exhibit superior performance in terms of convergence rate and learning efficiency. Table 1 validates the effectiveness of the RL algorithm in target search tasks within obstacle-free scenarios through task evaluation metrics. As shown in Table 1, SAC and TD3 achieve average reward values of 0.238 and 0.208, which represent a notable performance improvement compared to PPO’s 0.044. Specifically, SAC and TD3 achieve stable convergence after approximately 100 training episodes, with reward values eventually stabilizing around 0.2. This rapid convergence is attributed to their efficient policy exploration mechanisms. In contrast, PPO, constrained by its conservative policy update method, only converges after 400 episodes, and its average reward consistently remains below 0.2.

In terms of task execution performance, all three algorithms perform well, but there are differences in efficiency. Table 1 shows that SAC leads with a 99% success rate, and its task completion steps (159 steps) are reduced by 40% compared to PPO (265 steps). TD3 also achieves a 97% success rate, with an efficient performance of 160 steps. This indicates that SAC and TD3, through optimization strategies that maximize policy entropy, are able to effectively balance exploration and exploitation, thus quickly generating the optimal path in complex environments. Although PPO reaches a success rate of 87%, its higher task completion steps and loss rate (0.13) reflect limitations in adapting to dynamic environments.

### 5.3. Scenario 2: Target Search with Obstacle

**Figure 10:**
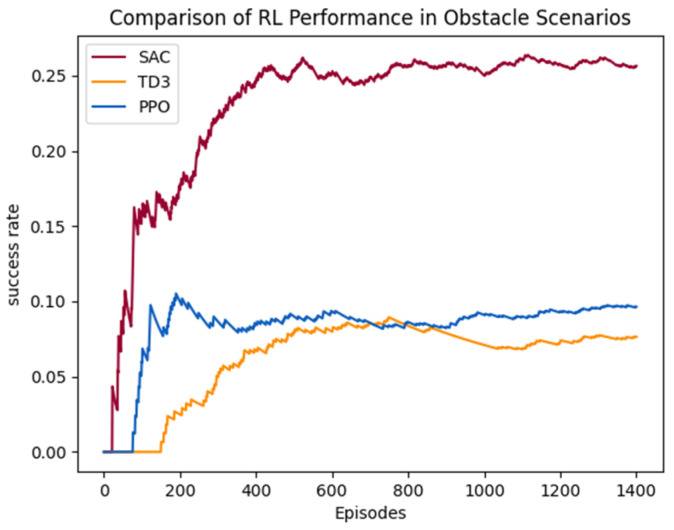
The task success rates of individual RL algorithms.

In a scenario with multiple obstacles, the UAV is initially trained using individual RL algorithms for obstacle avoidance and target search, but the training results are not ideal. As shown in Figure 10, the task success rates of PPO, SAC, and TD3 are all poor, with the overall success rate below 30%, and the success rates of PPO and TD3 even fall below 10%. This performance limitation primarily stems from two key factors: first, during the target search process, a single reinforcement learning algorithm struggles to effectively handle dynamic obstacle avoidance, especially due to inadequate real-time decision-making capabilities in complex environments; second, these algorithms have inherent flaws in the exploration–exploitation balance mechanism, which prevents the UAV from achieving optimal decisions between environmental exploration and target approach.

**Figure 11:**
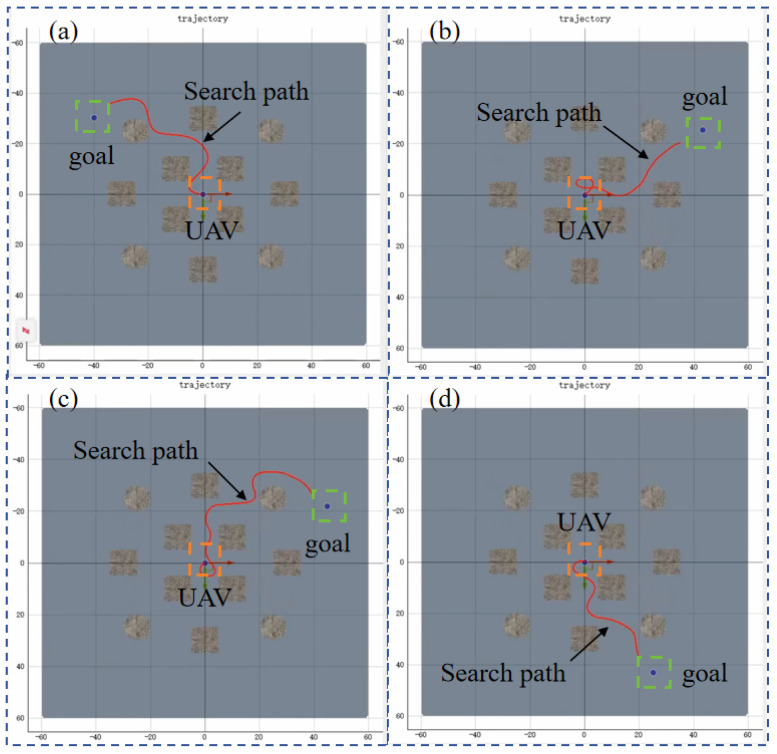
The search process for different target positions with obstacles. (**a**–**d**) are selected from four different episodes out of a total of 1400 episodes, targeting search tasks with randomly generated target positions in a complex obstacle scenario. The red lines represent the UAV’s search trajectory, starting from the center point, searching for the target while effectively avoiding obstacles. The orange square represents the UAV, and the green square represents the target.

**Figure 12:**
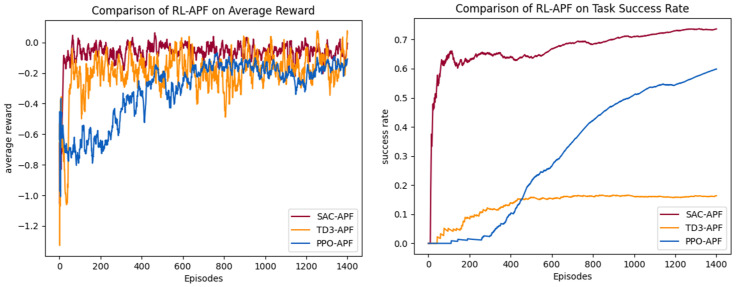
The training results of hybrid RL–APF algorithms in Scenario 2.

Therefore, the RL–APF hybrid algorithm structure is used for UAV training, incorporating APF-based obstacle avoidance control commands into the decision-making process. The training process is shown in Figure 11. The performance of PPO–APF, SAC–APF, and TD3–APF in the hybrid framework is compared. The average reward curves and task success rate curves are shown in Figure 12.

The comparative analysis of algorithm performance in Scenario 2 reveals breakthrough advantages of the hybrid SAC–APF architecture proposed in this paper. As shown in Table 2, SAC–APF achieves a 161% absolute improvement in success rate over the baseline SAC model, reaching 0.736 compared to the baseline value of 0.282, while its average reward is optimized from −0.238 to −0.063. This demonstrates that the APF framework effectively enhances environmental adaptability through synergistic optimization of potential field gradient guidance and maximum entropy policy, enabling efficient balance between target search and dynamic obstacle avoidance. The performance leap stems from a dual dynamic equilibrium mechanism: The local repulsive fields generated by APF accelerate gradient response for obstacle avoidance, while SAC’s maximum entropy policy ensures exploration efficiency in global target search. Their dynamic coupling successfully resolves the decision-making deadlock between obstacle avoidance and exploration in sparse-reward scenarios.

In contrast, the PPO–APF model exhibits significant performance trade-offs. Its success rate shows a 459% absolute improvement over baseline PPO, attaining 0.598 against the original 0.107, but the task completion steps surge from 124 to 340, corresponding to a 172% path efficiency loss. This phenomenon suggests optimization coupling barriers between PPO’s conservative policy update mechanism and APF’s real-time responsiveness. The TD3–APF hybrid presents an anomalous pattern: Its success rate improves by 95% relative to baseline TD3, climbing from 0.084 to 0.164, which exposes the deterministic policy’s susceptibility to local optimum traps under dynamic potential fields. This contrast highlights fundamental differences in policy gradient frameworks’ compatibility with artificial potential field guidance.

### 5.4. Discussion

This paper verifies the efficiency of three RL algorithms (PPO, SAC, and TD3) in target search tasks by comparing their performance in obstacle-free scenarios. However, in complex environments with obstacles, the performance of standalone RL algorithms significantly declines, primarily characterized by training instability, large reward fluctuations, and a higher task failure rate.

To address this issue, the RL–APF hybrid framework is proposed, optimizing both target search and obstacle avoidance, significantly improving task success rates. The experimental results show that SAC–APF within the hybrid framework achieves a success rate of 0.736, up from 0.282 in the basic SAC model, representing a 161% absolute improvement, validating the superiority of the hybrid framework in complex environments.

Compared to existing literature, the innovation and advantage of this study lie in the action-weighted fusion strategy that combines the global search capability of RL and the real-time obstacle avoidance feature of APF, achieving an efficient balance between target search and obstacle avoidance, which significantly enhances the task success rate.

## 6. Conclusions

In this study, a hybrid framework combining RL and APF is proposed to address the challenges of high computational complexity, poor real-time performance, and low efficiency in UAV target search tasks within complex environments. Through simulation experiments, the synergy between local obstacle avoidance and global search path planning for UAVs is validated. The contributions of this research are as follows. Firstly, a task scenario and training environment specifically for UAV target search are constructed. Secondly, by integrating RL and APF, a global-local strategy is developed, significantly improving the success rate of target search tasks in complex environments. Thirdly, a comprehensive comparison of the performance differences between the hybrid framework and standalone RL algorithms is conducted through training and analysis. The experimental results show that the proposed hybrid framework outperforms standalone RL-based approaches in terms of target search efficiency and obstacle-avoidance performance, validating its potential applicability to UAV autonomous navigation tasks. Although this study has achieved certain results, further work is still needed in the future. Future research directions include: first, testing the hybrid framework in real-world environments to verify its performance and robustness; second, optimizing the hybrid framework to improve computational efficiency and adaptability; third, integrating the hybrid framework into actual UAV hardware, considering factors such as sensor noise and computational resource limitations; and finally, extending it to dynamic obstacle environments to enhance adaptability.

## Figures and Tables

**Table 1:** Task evaluation metrics for Scenario 1.

Metric	PPO	TD3	SAC
average reward	0.044	0.208	0.238
loss rate	0.13	0.03	0.01
success rate	0.87	0.97	0.99
completion steps	265	160	159

**Table 2:** Task evaluation metrics for Scenario 2.

Metric	PPO	TD3	SAC	PPO–APF	TD3–APF	SAC–APF
average reward	−0.217	−0.168	−0.238	−0.306	−0.194	−0.063
loss rate	0.893	0.916	0.718	0.402	0.836	0.264
success rate	0.107	0.084	0.282	0.598	0.164	0.736
completion steps	125	102	111	340	475	153

## Data Availability

The data presented in this study are available on request from the corresponding author.
